# Rapid IoT Prototyping: A Visual Programming Tool and Hardware Solutions for LoRa-Based Devices

**DOI:** 10.3390/s23177511

**Published:** 2023-08-29

**Authors:** Juan José López, Paula Lamo

**Affiliations:** 1 Arduinoblocks and Salesianos Juan XXIII Alcoy, 03804 Alcoy, Spain; arduinoblocks@gmail.com; 2 Escuela Superior de Ingeniería y Tecnología, Universidad Internacional de La Rioja, 26006 Logroño, Spain

**Keywords:** IoT, rapid prototyping, education, LoRa, LoRaWAN, Arduinoblocks, ESP32

## Abstract

LoRa technology has gained popularity as one of the most widely used standards for device interconnection due to its ability to cover long distances and energy efficiency, making it a suitable choice for various Internet of Things (IoT) monitoring and control applications. In this sense, this work presents the development of a visual support tool for creating IoT devices with LoRa and LoRaWAN connectivity. This work significantly advances the state of the art in LoRa technology by introducing a novel visual support tool tailored for creating IoT devices with LoRa and LoRaWAN connectivity. By simplifying the development process and offering compatibility with multiple hardware solutions, this research not only facilitates the integration of LoRaWAN technology within educational settings but also paves the way for rapid prototyping of IoT nodes. The incorporation of block programming for LoRa and LoRaWAN using the Arduinoblocks framework as a graphical environment enhances the capabilities of the tool, positioning it as a comprehensive solution for efficient firmware generation. In addition to the visual tool for firmware generation, multiple compatible hardware solutions enable easy, economical, and stable development, offering a comprehensive hardware and software solution. The hardware proposal is based on an ESP32 microcontroller, known for its power and low cost, in conjunction with an RFM9x module that is based on SX127x LoRa transceivers. Finally, three successfully tested use cases and a discussion are presented.

## 1. Introduction

The Internet of Things (IoT) has revolutionized how people interact with the world, connecting everyday objects and enabling an ecosystem of interconnected devices [1]. In this scenario, Long-Range (LoRa) technology emerges as a leading option for IoT applications that require long-distance and efficient wireless communication [2]. Patented by the Semtech company, LoRa uses the Direct Sequence Spread Spectrum (DSSS) modulation technique for data transmission and the Chirp Spread Spectrum (CSS) technique to spread the signal across the frequency spectrum [3,4,5]. The CSS technique sends a Chirp signal that varies in frequency linearly over time. Upon receiving the Chirp signal at the receiver, a mathematical process is carried out to reconstruct the original signal. Since this signal is spread out across the frequency spectrum, it becomes less susceptible to interference and noise, making it possible to transmit it over longer distances and detect it more accurately. Its ability to provide wide geographic coverage and allow efficient long-distance communication is achieved thanks to its low power consumption and high sensitivity. Regarding costs, LoRa adopts an open-source Low Power Wide Area Network (LPWAN) approach, facilitating cheaper deployment and expansion. In contrast, similar technologies, such as Sigfox, require specific network infrastructure and subscriptions, which can result in higher costs for large-scale deployments [6,7].

Here, LoRaWAN emerges as a network communication protocol for IoT applications based on LoRa technology. It allows the connection of IoT devices to a centralized network and enables bidirectional data transmission, facilitating the integration of sensors and actuators in various applications [8]. One of the main advantages of LoRaWAN lies in its ability to provide security and control over data communication [9]. The encryption protocol built into LoRaWAN ensures data protection between devices and the network, a crucial feature in applications involving sensitive or confidential information. In addition, its decentralized architecture allows for a higher degree of control and flexibility in device configuration and management, thus simplifying monitoring and control of data flow in the network [10].

The successful implementation of LoRa connectivity with the LoRaWAN protocol requires the provision of an application server and gateway infrastructure [11,12,13,14]. Interested parties can use The Things Network (TTN) service or implement their LoRaWAN infrastructure using The Things Stack (TTS) server and custom gateways. The choice between these approaches will depend on the specific needs and resources of each project. TTN could be a suitable choice for fast and cheap solutions. A proprietary LoRaWAN server could be preferable in projects with particular coverage, security, or personalization requirements. In any case, adopting LoRa technology and deploying LoRaWAN networks present challenges for those new to IoT or wireless technology [15]. Configuring LoRa nodes, programming firmware, and integrating with a LoRaWAN network infrastructure can be complicated and daunting [16], which can be demotivating for beginners or students. In addition, there is a shortage of tools to develop LoRa prototypes in educational environments due to their state-of-the-art nature. Technical complexity and the need for solid hardware and software knowledge make it difficult for less experienced users or educational settings to adopt. Although there are modules that facilitate LoRa connections with microcontrollers via SPI, the relevant information is scattered. In many cases, the complex and difficult-to-access documentation of the manufacturer makes it difficult to understand [6,7]. The libraries to control these modules are usually in C++, making them difficult to understand and use. Some Arduino models, such as the MKR1310, seek to simplify LoRa prototyping [17]. However, their limited availability and high cost can be disadvantages. On the other hand, ESP32-based modules with LoRa connectivity, developed by Heltec, are popular [18]. Incorporating OLED screens, these modules are programmed in C++ with Arduino and the libraries of the manufacturer. Despite more affordable and simple solutions, none focuses on modular connection, which is effective in educational settings by standardizing the connection of sensors, actuators, and peripherals, avoiding prototyping boards and complex connections. All current solutions require textual programming in C++ for Arduino.

In this context, visual support tools for rapid prototyping of LoRaWAN-based IoT devices are playing a crucial role [19]. One prominent example is Arduino [20,21], an open-source hardware and software platform that allows users, even those without programming experience, to quickly prototype IoT devices using a graphical interface. In addition, the Raspberry Pi [22] has revolutionized the way IoT and programming are taught, allowing users to create more complex and connected projects. These platforms provide a solid foundation for developing IoT solutions, fostering creativity and innovation in different fields such as home automation, smart agriculture, environmental monitoring, and urban infrastructure management [23]. These initiatives seek to simplify the development process for IoT devices, providing a more accessible and user-friendly experience for students, beginners, and professionals looking to enter the world of IoT, as shown in ref. [24]. In line with these trends, developing a visual support tool for rapid prototyping of LoRaWAN-based IoT devices presents a valuable opportunity to bring LoRa technology closer to educational and research environments [25]. The combination of an intuitive graphical environment, compatible hardware, and a real-time LoRaWAN infrastructure makes it easy to experiment and learn, which can significantly impact the growth and adoption of IoT solutions globally [26]. Therefore, this paper presents the development of a visual support tool for rapidly prototyping IoT devices based on LoRa technology and the LoRaWAN protocol. It focuses on facilitating the access and programming of LoRa nodes through an intuitive graphical interface, allowing students, beginners, and professionals to create prototypes easily. The tool uses the Arduinoblocks block programming environment, based on the Google Blockly framework, to generate the necessary firmware. Moreover, this work chooses the ESP32 STEAMakers board, based on the ESP32 with modular connectivity, for educational environments because it allows programming in C++ with the Arduino IDE, MicroPython, and blocks with Arduinoblocks. The project included the RFM95W module with the SX1276 chip, widely used in LoRa and LoRaWAN solutions. It provides LoRa connectivity to microcontrollers via SPI. Likewise, three successful use cases are discussed, such as a weather station and bidirectional forwarding of LoRa data with an MQTT server over WiFi, GPS vehicle tracking, and urban lighting control, demonstrating the effectiveness and usefulness of this tool in various IoT applications.

## 2. Integral Solution for Rapid Prototyping

In the development of this work, two different functional parts have been identified. The first part focuses on developing IoT nodes based on LoRa technology, programmed using an intuitive visual tool (Arduinoblocks). This stage involves creating and configuring devices that use LoRa technology for data transmission and communication. The visual tool simplifies the programming and control of the nodes, facilitating their configuration and operation. Hence, the second part focuses on the architecture used by LoRa nodes in test or production environments. This architecture manages the communication between the nodes and processes and stores the transmitted data. Its complexity and scalability vary depending on the deployment environment and the needs of the project.

For example, in a point-to-point configuration, two LoRa nodes establish direct communication, one acting as a sender and the other as a receiver. This configuration allows the efficient exchange of information over long distances, taking advantage of LoRa modulation technology with wide coverage and penetration capacity [27]. The receiving nodes decode the LoRa signal, guaranteeing the successful reception of the data. In contrast, another scenario involves deploying LoRa nodes within a common coverage area, allowing one-to-many communication. It can be through a master node and slave nodes or a network of nodes with sensors and a central receiver node. A protocol establishes the origin and destination of the data, including a header in the message to address the information correctly. Here, the LoRaWAN protocol, designed for low-power wide-area networks, is recommended for secure communication [28]. Configuring a communication gateway that acts as an access point between the LoRa nodes and the LoRaWAN network is required. A LoRaWAN-compliant application server, such as TTS, manages the network, authenticates the nodes, routes the messages, and manages the transmitted data.

Regarding software development, three main components are identified:

1. Visual block programming: This component provides an intuitive interface for configuring the logic of IoT LoRa nodes.
2. Automatic code generation: The corresponding code is automatically generated in a specific programming language, such as C++ or Arduino.
3. Compilation and uploading: The code is compiled and uploaded to the ESP32 board via USB. This architecture effectively combines the user-friendliness of visual programming with the adaptability and performance of generated code, streamlining the efficient design and setup of IoT LoRa nodes.

### 2.1. Hardware Prototype

For the developed prototype, hardware consisting of the ESP32 STEAMakers board [29] and LoRa RFM95W module [30] based on the Semtech SX1276 chip was selected. This module uses the Serial Peripheral Interface (SPI) communication bus for data transfer and requires additional pins for complementary signal management. The pins of the RFM95W module have been configured to be compatible with the SPI pins of the ESP32 microcontroller. The pins assigned to the VSPI bus are used to establish communication with the RFM95W module.

One of the features of the ESP32 STEAMakers that makes it attractive for this project and why it has been chosen is its modular connection. This feature duplicates the input/output pins and the power pins (V and GND), which is extremely useful in both prototyping and educational settings. The modular design allows users to quickly develop and expand their projects by facilitating the seamless integration of various components, promoting a fluid and dynamic prototyping experience. This strategic arrangement streamlines the assembly of complex configurations. It improves the platform’s accessibility and versatility for educational purposes, allowing students to gain hands-on experience in a multifaceted learning environment.

There are several formats for connecting these modules to a microcontroller: the Arduino shield, the RFM9x, and the modular with pins. The Arduino shield format allows for easy connection by placing the expansion board on top of the ESP32 STEAMakers controller board. However, this prevents access to the modular connection pins, requiring breadboards to connect additional sensors or actuators. Using this commercial shield implies losing the modular connection functionality offered by the ESP32 STEAMakers. For their part, some modules include an adapter plate to access the pins. A typical example is the RA-01 module, which uses the SX1278 chip and provides access to the SPI and DIO-0 pins but only supports LoRa mode, not LoRaWAN. At the same time, the RFM9x module format allows the design of a simple board to directly connect the module to the pins of the ESP32 STEAMakers board without losing access to the other pins and taking up little space. For these advantages, it is the adopted solution. With this proposal, an exchange is guaranteed between the long-range communication capabilities offered by LoRa and the comfort and adaptability intrinsic to the modular framework of the ESP32 STEAMakers.

**Figure 1:**
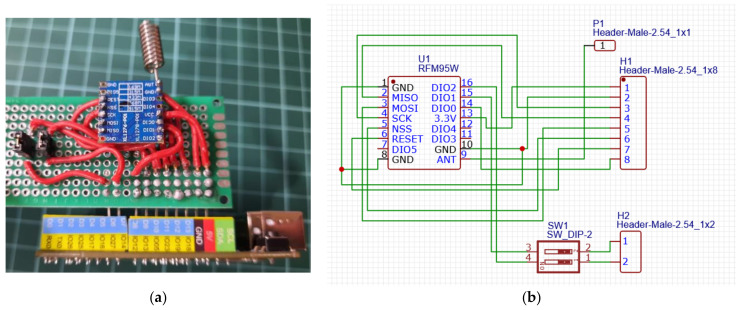
First experimental setup test: (**a**) prototype and (**b**) connection diagram.

**Figure 2:**
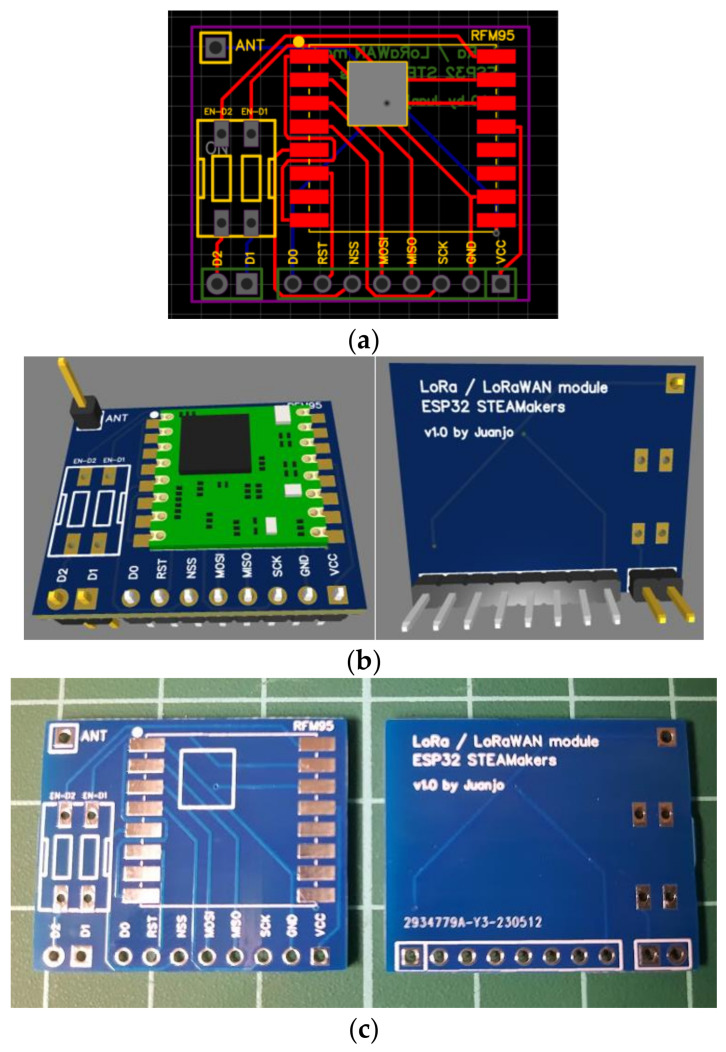

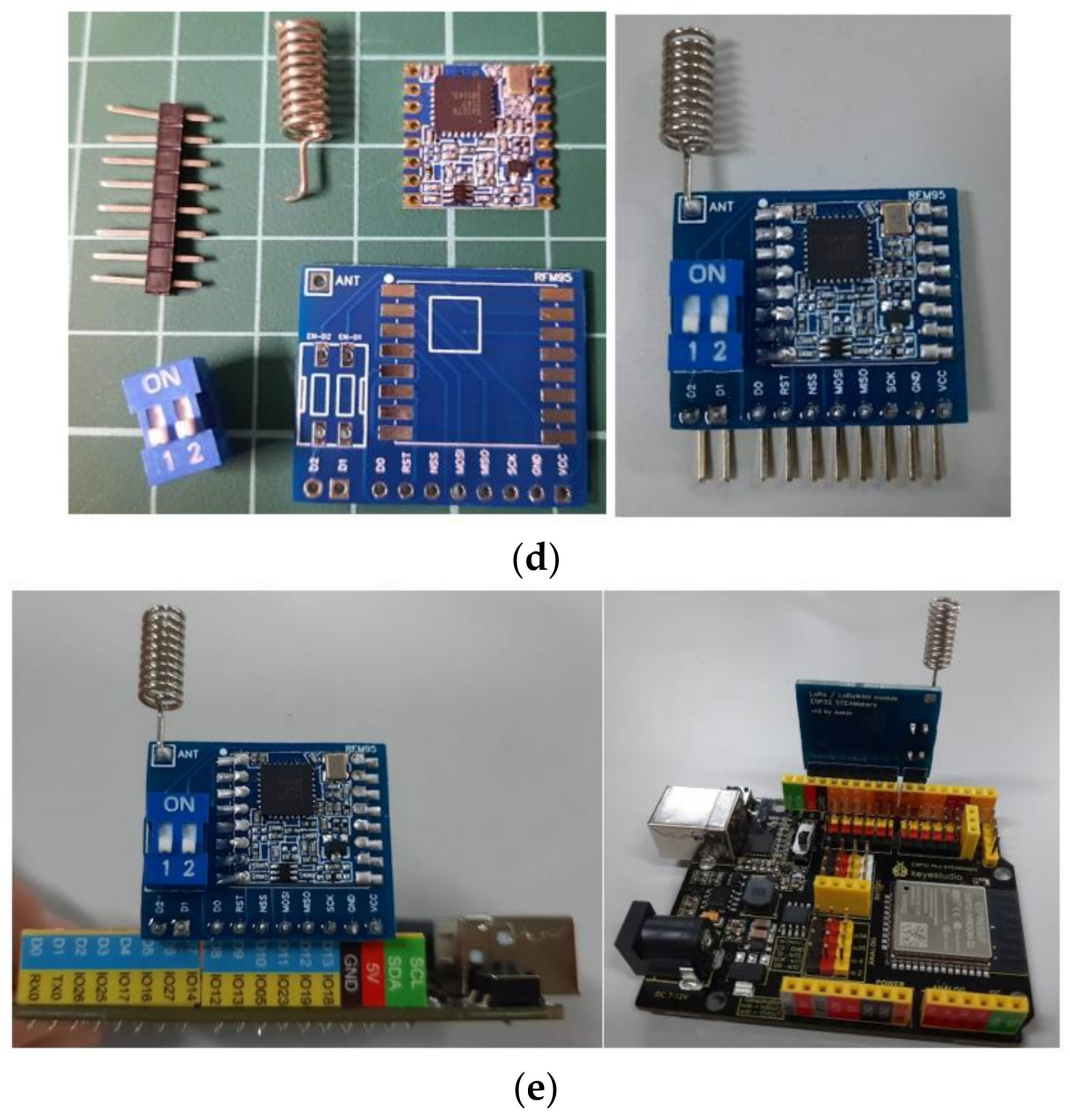
Final prototype: (**a**) PCB-designed adapter module; (**b**) 3D simulation of the designed adapter module; (**c**) manufactured PCB; (**d**) components and PCB before and after soldering; and (**e**) module connected to the ESP32 STEAMakers board ready to use.

To facilitate connections and accommodate the pin spacing, a board was developed, as the RFM95W module does not use the standard 2.54 mm pin spacing commonly used on prototyping boards and other chips. A prototype was created using a generic board and wiring connections (Figure 1). After verifying its feasibility, a second prototype, shown in Figure 2, was developed using a printed circuit board (PCB) designed to adapt the RFM95W module and allow a simple connection with the ESP32 STEAMakers board using the EasyEDA tool [31]. Based on the PCB design, five breadboards were manufactured to evaluate its functionality, ensure correct and reliable connections, and adapt to the standard distance between pins with the custom-designed PCB.

### 2.2. Software Development

**Figure 3:**
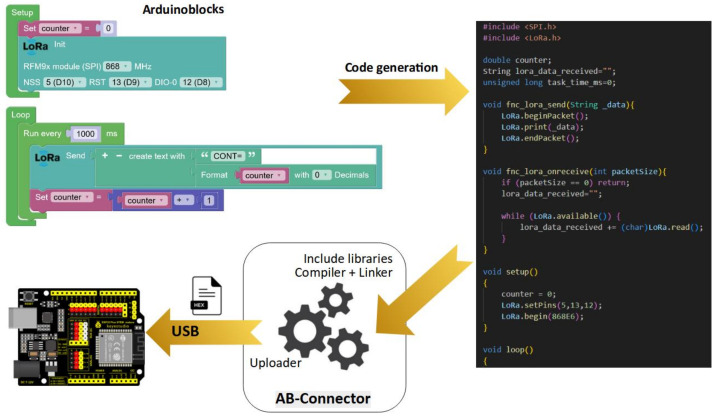
Overall schematic of the proposed code generation process in the tool.

The visual tool used in this work is Arduinoblocks [32], a block-based visual programming environment used to program Arduino boards. It provides an intuitive graphical interface that allows creating and programming projects using the Arduino programming language without needing to write code, according to the schematic in Figure 3. Instead of writing lines of code, Arduinoblocks users can drag and drop predefined programming blocks to create their program flow. These blocks represent Arduino instructions and functions, such as reading sensors, controlling actuators, performing calculations, and making logical decisions. In this way, Arduinoblocks simplify the programming process by hiding the complexity of low-level code and allowing users to focus on the logic of their project [33]. It is a handy tool for beginners, students, and those who want to learn programming and electronics in an accessible way. Once the visual program has been created using Arduinoblocks, it can be transferred and run on an Arduino board to control physical devices and do interactive projects. However, although Arduinoblocks is compatible with different Arduino boards and offers an easy way to program different functions, until the completion of this project, LoRa- and LoRaWAN-compatible libraries were not available.

To solve the latter, the capabilities of the platform have been expanded, enabling the functionalities that allow the use of the hardware developed in this project. In this way, the “sandeepmistry” library [34], compatible with SX127x-based transceivers, is used for communication in LoRa data frames. This widely recognized and standardized library simplifies sending and receiving data frames through LoRa modules. Furthermore, it follows a structure and nomenclature similar to the library used by the Arduino model MKR1310.

To manage communication with the SX1276 chip and establish LoRaWAN connectivity in the development of the project, the “TTN-ESPttn-esp32” library [35] has been used. This library is based on the LMIC library developed by IBM and adapted for use on various platforms. In addition, it simplifies the management of the LoRaWAN connection. It allows easy adaptation to different SX127x chip models and the pins used, depending on the shield configuration or the connections between the microcontroller and the SX1276 chip. In addition, the configuration of the connection pins and the LoRaWAN parameters for authentication through activation over-the-air (OTAA) or activation by personalization (ABP) is performed, and a callback function is defined to process the data received.

**Figure 4:**
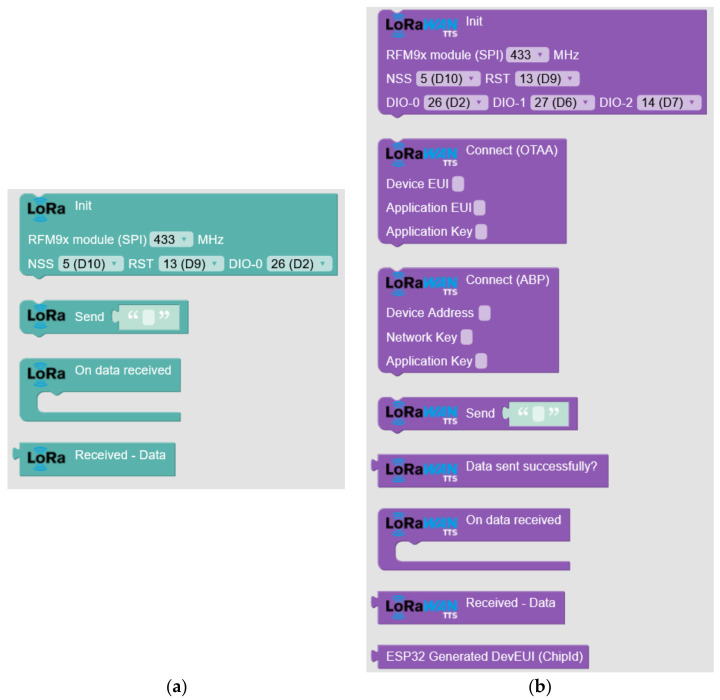
Appearance of the new blocks configured in Arduinoblocks: (**a**) LoRa blocks and (**b**) LoRaWAN blocks.

To integrate these libraries in Arduinoblocks, it is necessary to develop the new blocks that will be used with Blockly from Google [36] using the C++ language for Arduino. This task is divided into two stages: definition and implementation. The first is the definition of each block and the implementation of specific code generators. In the definition stage, the parameters and characteristics of each block are set, such as the number of entries and whether it returns a value. In the implementation stage, code generators convert the blocks into source code in the desired language. During the process, Blockly walks through the blocks used in the program and combines them to form the complete code. This includes control structures, function calls, and additional code necessary for the proper operation of the program. The LoRa-related blocks (Figure 4a) include the functions “Start” to configure the connection with the RFM9x (SX127x) module, “Send” to transmit LoRa data frames, “Received Data Event”, which is triggered when receiving data via LoRa, and “Received Data” to access the received data. In the blocks related to LoRaWAN (Figure 4b), there are “Init” to configure the connection with the RFM9x module, “ConnectOTAA” to establish the connection through OTAA authentication, “ConnectABP” to configure the connection through ABP authentication, “Send” to send LoRaWAN data frames, “Data sent successfully?” to verify the status of the last shipment, “On data received” that is executed when receiving data via LoRaWAN, “Received Data” to access the data received, and “ESP32 DevEUI” that generates a valid DevEUI to register in the application TTN from the MAC of the ESP32 microcontroller.

The algorithm and codes used to define LoRa and LoRaWAN blocks in Figure 4 can be seen in the repository of Data Availability Statement.

**Figure 5:**
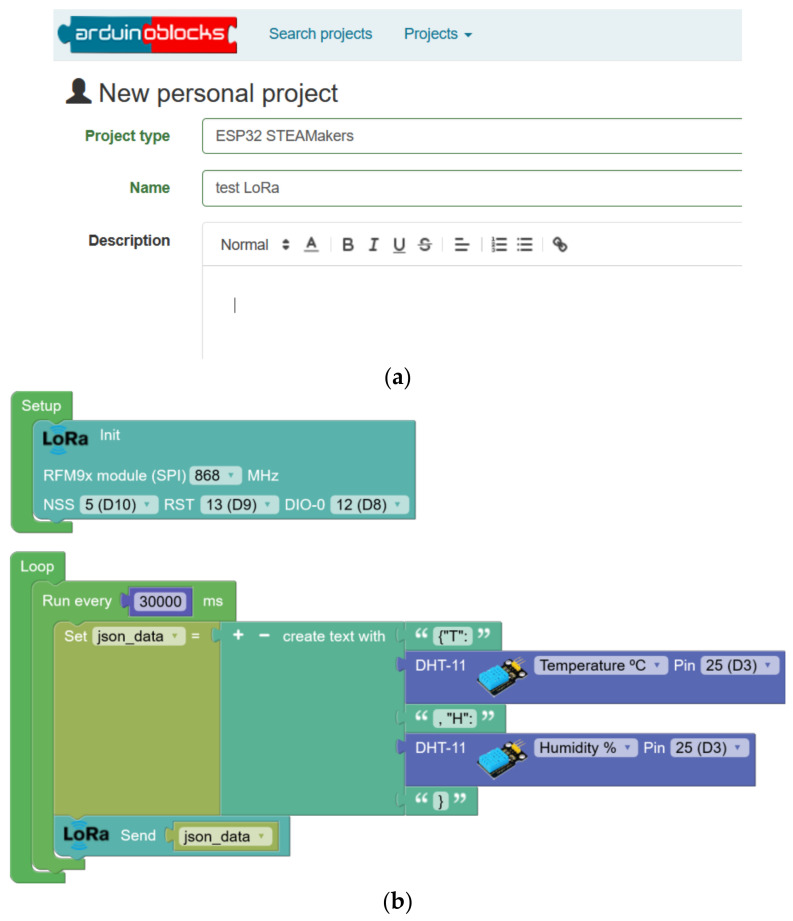
Appearance of Arduinoblocks: (**a**) home screen for project creation and (**b**) work screen for project construction.

Once the development, testing, and verification of the programming blocks for LoRaWAN have been completed, they are integrated into the Arduinoblocks platform [37] to create an integrated environment that allows users to build complete programs that involve LoRa technology, as well as other components of hardware and functionalities available on the platform. The developed LoRa and LoRaWAN blocks are now available in the toolbar of ESP32 STEAMakers-type projects on the platform. To start programming, users must log in (Figure 5a) and create a project (Figure 5b).

The Arduinoblocks platform is founded upon an online tool that can be executed within contemporary web browsers such as Chrome, Firefox, or Opera. Nonetheless, the execution of the application responsible for compilation and uploading is imperative, serving as an intermediary between the web browser and the hardware. This application, called AB-Connector, is accessible across various operating systems, including Windows, Linux, MacOS, and ChromeOS. In scenarios where an alternate system is employed for web browsing, AB-Connector configuration on a remote device becomes viable, facilitating remote hardware deployment. For instance, one can utilize an Android tablet to connect to the AB-Connector application hosted on a device within the local network, aligning with the designated operating systems.

## 3. Tested Use Cases

For the testing of the equipment built, tests were carried out using the LoRaWAN network implemented by the company IoTsens [38] in the city of Alcoy (Spain). This network provides coverage for the entire city through the installation of multiple gateways. The platform is based on the TTS server, which is managed by the implementing company and the city council. Three developed examples are shown in this section.

### 3.1. Weather Station with Temperature and Humidity and Bidirectional Forwarding of LoRa Data with an MQTT Server over WiFi

**Figure 6:**
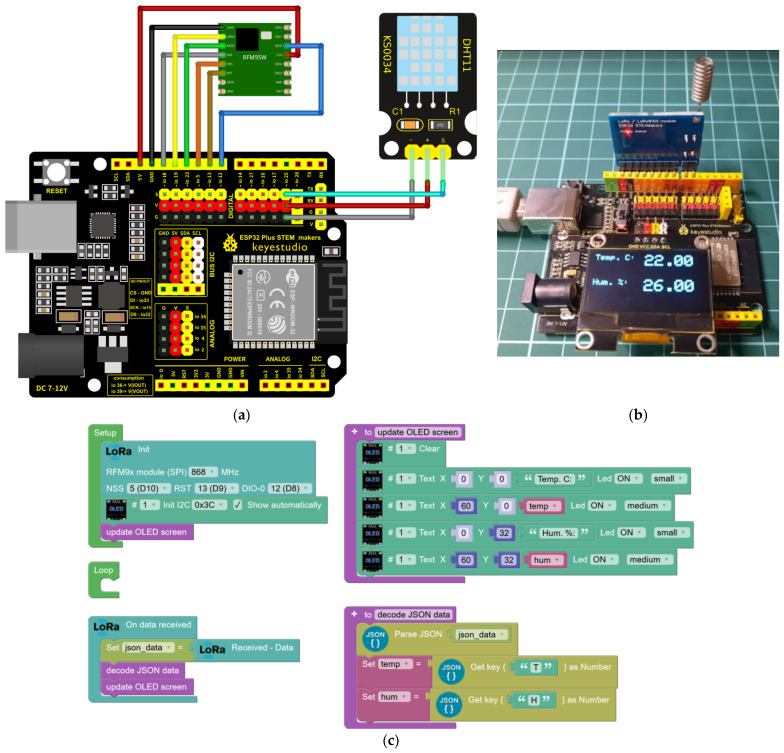
Experimental setup dedicated to a weather station with temperature and humidity: (**a**) connection diagram; (**b**) prototype of the LoRaWan node; and (**c**) block programming for a data reception and visualization program.

A point-to-point remote communication scenario is proposed. Here, a sending device (consisting of ESP32 STEAMakers, an RFM95W-SX1276 module, and a DHT-11 sensor) periodically sends the temperature and humidity values (Figure 6a,b). This data will be encoded in JSON format before transmission, which makes it easy to organize and analyze individual data on the receiving device. On the receiving side (consisting of ESP32 STEAMakers, an RFM95W-SX1276 module, and an OLED display with an I2C connection), the data will be received and decoded for display on an OLED display.

The sending node program reads the sensor data and encodes it in JSON format (Figure 5c). The transmission is performed every 30 s through the LoRa module. In the system schematic, the DIO-1 and DIO-2 pins are not used in LoRa mode, and their representation in the diagram is omitted. Moreover, it is important to ensure that the micro-switches are in the off position for the correct operation of the system.

**Figure 7:**
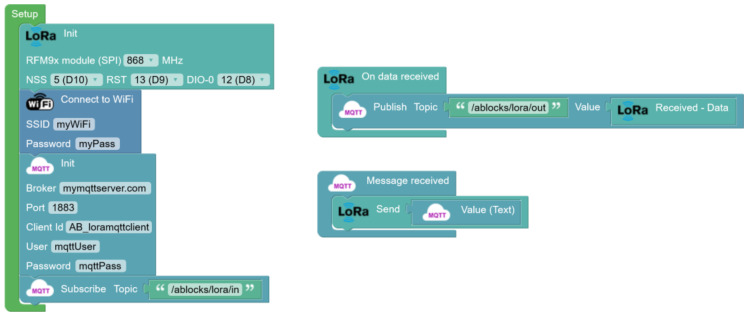
Block programming for a data reception and visualization program for the bidirectional forwarding of LoRa data with an MQTT server over WiFi.

Next, an intermediary connection is established between a LoRa device network and an MQTT server (Figure 7), aimed at facilitating bidirectional data transmission among devices and creating a communication infrastructure within a building environment utilizing LoRa technology. An ESP32 board will be employed to achieve this functionality, which will link to a WiFi network to establish connectivity with the MQTT server. This device is the central node, responsible for mediating data transfer between devices and the MQTT server in both directions. Additionally, this setup allows seamless integration of the home automation network with other systems, such as home management applications [39], all of which leverage the MQTT protocol for communication.

In one aspect, the ESP32 board captures data emitted by LoRa devices within its coverage radius, forwarding it via the WiFi network to the MQTT server. Simultaneously, the ESP32 board will subscribe to the topic on the MQTT server. Any data received under this topic through MQTT will be transmitted back to surrounding devices via LoRa technology. The implementation of this gateway streamlines the interoperability and integration of various communication technologies, allowing for centralized and effective device management and the ability to harness the unique qualities and advantages inherent in each specific communication technology.

### 3.2. Vehicle Location Via GPS

**Figure 8:**
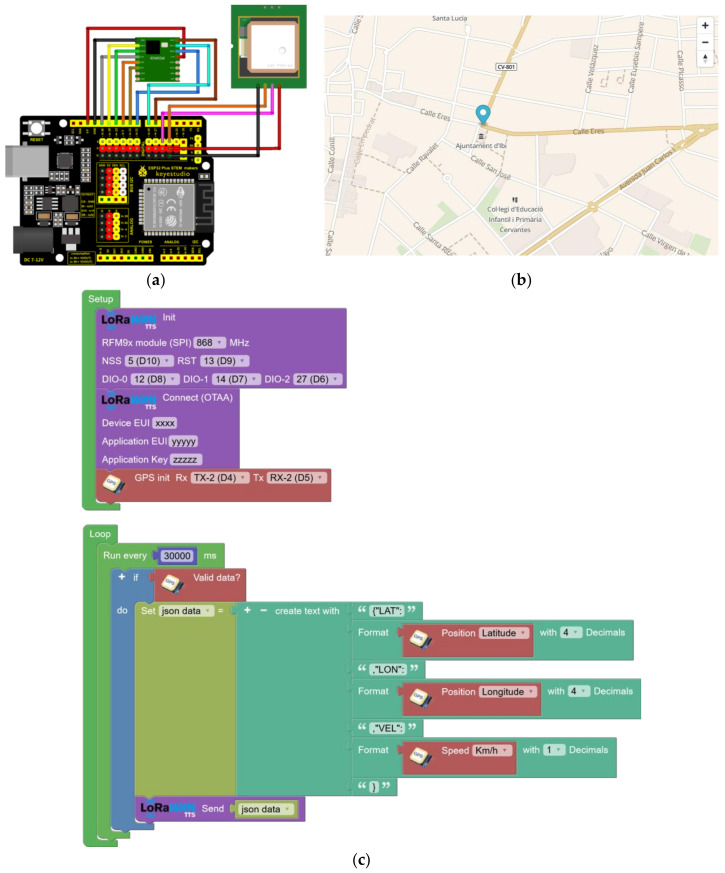
Experimental setup dedicated to vehicle location via GPS using LoRaWAN: (**a**) connection diagram; (**b**) example of visualization on the map of the GPS data; and (**c**) block programming for sending the GPS geolocation via LoRaWAN every 30 s.

An example of a track-and-trace node implementation using a GPS is presented. The goal is to track the location of a vehicle (equipped with an ESP32 STEAMakers Board, an RFM95W-SX1276 module, and a NEO6/7 GPS module) in a city with a LoRaWAN infrastructure that covers the entire metropolitan area (Figure 8a). The data received includes latitude, longitude, and speed, which allows the position of the vehicle to be represented on a map.

The track and trace node has a GPS module that captures the vehicle’s location information. Using LoRaWAN, latitude and longitude data is transmitted wirelessly over the network to a central server. This central server, connected to the city’s LoRaWAN infrastructure, receives the data from the track and trace node. Furthermore, it processes and stores the information in a database or forwards it via MQTT to an application. The complete program is presented in Figure 8c.

To visualize the position of the vehicle on a map, a web or mobile application can be developed that accesses the central server’s database. Using the received latitude and longitude data, the app displays the vehicle’s location in real-time on an interactive map (Figure 8b). This solution allows precise tracking and location of the vehicle in real-time in the metropolitan area using LoRaWAN technology and location information provided by the GPS module.

### 3.3. Monitoring and Remote Control of the Lighting System

**Figure 9:**
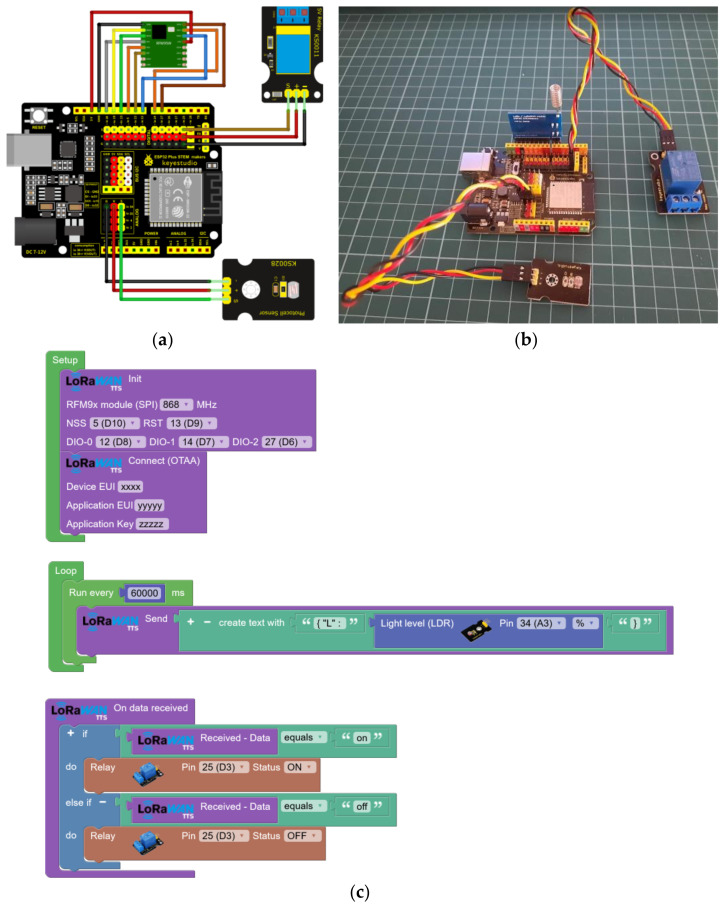
Experimental setup dedicated to streetlight control and ambient light monitoring: (**a**) connection diagram; (**b**) prototype of the LoRaWan node; and (**c**) block programming to send ambient light level every 60 s and remote control of the relay.

In this use case, the automation of street lighting using an ESP32 STEAMakers, an RFM95W (SX1276) module, a relay module for an AC/10A load, and an analog LDR sensor (Figure 9a,b) is being proposed. Each streetlight is equipped with an IoT node that monitors the ambient lighting level using an LDR photocell on top. In addition, the node controls the switching on and off of the streetlight through a relay that regulates the electrical supply. Using IoT nodes with LoRaWAN connectivity allows wireless data transmission at the lighting level to a central management platform.

The implementation involves installing IoT nodes on each streetlight equipped with an LDR photocell to capture information about ambient lighting. This information is sent periodically through LoRaWAN connectivity to a central platform (Figure 9c). In addition, the TTS application server makes receiving and sending data using different protocols, such as HTTP or MQTT, possible. This allows users to create a web panel to monitor the ambient light level of a network of streetlights and send on or off commands to each node.

## 4. Discussion

This project has involved the combination of various technologies. The ESP32 microcontroller has been used as a base, providing processing capabilities and connectivity for LoRa technology and the LoRaWAN protocol. The Arduinoblocks development environment and third-party libraries have made it possible to compile and load the firmware, manage communication, and implement the LoRaWAN protocol. The Blockly JavaScript library has been used to create a block-based visual programming environment, facilitating the intuitive programming of devices. Node.js has provided an efficient runtime for the visual programming environment. Specialized PCB design tools such as EasyEDA have been used to design and manufacture an ESP32 STEAMakers-compatible prototyping board. In addition, an instance of The Things Stack has been installed on a cloud server to manage the LoRaWAN environment, including device management and data routing. The combination of these technologies has been essential for the successful development of this work, allowing the interconnection of LoRa devices and the deployment of a LoRaWAN infrastructure.

### 4.1. Development Environments for Design Projects

Visual programming tools are an option for those who want to learn programming or build apps without prior programming knowledge. These tools facilitate the understanding of programming concepts and allow users to create interactive and creative projects simply and visually, avoiding syntax problems. Developing a visual tool is hard work, so many development environments rely on pre-built frameworks that implement block creation, code generation, and other tools.

Some of the environments that support the development of a custom editor for visual programming are Scratch [40], a tool for developing small applications and games, and Snap! [41], which offers advanced programming features. These open-source frameworks allow for customization and tuning to suit specific needs. Blockly is a framework for creating custom visual programming environments and is used in tools like MIT AppInventor [42]. In contrast, MakeCode [43], developed by Microsoft, focuses on Micro:bit hardware and enables visual block programming. In addition, Node-RED [44] is a visual programming environment that connects systems and develops solutions by connecting nodes. However, in this work, Blockly has been chosen as the basis for developing the visual tool since it is highly customizable and allows integration with the Arduinoblocks platform, which is also based on Blockly. This ensures greater consistency and compatibility in the visual programming environment used in the project.

Arduinoblocks facilitates the visual development of firmware for the most prevalent microcontrollers within the Arduino environment, such as Arduino UNO, Nano, or Mega. These microcontrollers often exhibit limited processing power in relation to contemporary development requirements. As an alternative, the ESP32 microcontroller has emerged as an affordable and high-performance solution, finding widespread application across myriad prototyping and development contexts. Notably, this microcontroller boasts robust capabilities while maintaining a budget-friendly cost, rendering ESP32-based prototyping development boards remarkably economical. Arduinoblocks extends its support to the ESP32, encompassing a multitude of its functionalities, particularly those associated with Bluetooth and WiFi connectivity.

### 4.2. Educational Kits for LoRaWAN

In an educational environment, several kits that incorporate LoRa connectivity and the LoRaWAN protocol are available. These kits allow students to develop their own IoT solutions. Some examples include “The Things Network Starter Kit” [45], which provides a LoRaWAN gateway and end devices for building IoT projects. Another kit is the “Pycom IoT Kit” [46], which includes a LoRaWAN-compatible Pycom LoPy4 board, sensors, and electronic components for building IoT solutions. The “LoRaWAN Discovery Kit” [47] is also available, consisting of an STM32 board, a LoRaWAN module, and various sensors and actuators. This kit comes with a detailed guide for teachers to teach students about LoRaWAN and how to build IoT projects. In addition, the “RAK Wireless WisBlock Kit” [48] offers a LoRaWAN gateway and LoRaWAN-enabled WisBlock boards, along with sensors and actuators, so students can develop their own IoT solutions. In addition, the “Edukit LoRaWAN” [49] comprises a LoRaWAN-compatible board and several sensors and actuators. This kit includes a detailed user guide that helps students learn and use LoRaWAN technology. All these kits are valuable resources to facilitate the introduction of LoRa connectivity and the LoRaWAN protocol in the educational environment, allowing students to explore and experiment with the Internet of Things.

Compared with these kits, the comprehensive proposal of this work presents students with a complete and economical working environment that guarantees the compatibility of the components used with the Arduinoblocks platform to carry out block programming and introduce IoT technologies to students or beginners who have no programming knowledge. This allows the teacher to focus on teaching technology over and above the programming structures, guaranteeing the autonomy of the students, and developing prototypes in less time.

### 4.3. Scope and Limitations

This work facilitates the introduction of LoRa technology and the LoRaWAN protocol in the educational environment, especially in classrooms, through a simple and effective didactic approach. Students are required to have prior knowledge of basic electronics and programming, with fundamental notions of electricity, electronics, and basic programming concepts. They do not need experience in a specific programming language, but they are expected to be familiar with the basic concepts and algorithms associated with programming. The selection of affordable and accessible hardware components is emphasized to facilitate their acquisition in the form of kits for students. However, the main limitation is the availability of adequate infrastructure for interconnection through LoRaWAN, which is not available in all educational centers or cities and is not accessible to all students. The alternative of purchasing a LoRaWAN gateway and configuring it on the public TTN network to provide access to this open global infrastructure is being considered. However, the limitations imposed by the free option must be considered.

In addition, communication through LoRa technology, operating within a shared frequency range, can be intercepted by any compatible radio device operating on the same frequency. Given this limitation, Arduinoblocks must incorporate a data encryption system to ensure the confidentiality of communications between devices.

Furthermore, several blocks are currently under development within Arduinoblocks to enable the configuration of advanced parameters for LoRa communication. These pending blocks include adjusting transmission power, spreading factor, sync word value, and other advanced settings currently set to the default standard. These finely adjustable settings are crucial for optimizing LoRa communication operations according to the specific requirements of each scenario and project, offering greater flexibility and adaptability to various applications and needs. The successful implementation of these blocks within Arduinoblocks will equip users with an even more versatile and potent tool for designing and executing projects that utilize LoRa technology, supporting security and customization at every stage of the development process.

### 4.4. Future Works

Development is underway to introduce features encompassing data encryption and decryption, ensuring data security in LoRa communications. By doing so, the transmitted data utilizing LoRa (not LoRaWAN), which could be intercepted by any LoRa radio, will remain unintelligible to recipients without the correct decryption key. This ongoing functionality implementation aims to enable straightforward and secure data exchange, bolstering the ease of secure data communication while utilizing LoRa technology.

Moreover, configuring advanced LoRa communication parameters will incorporate these encryption and decryption functionalities. This holistic approach to incorporating advanced parameters and security features will empower Arduinoblocks users with an all-encompassing toolset for the seamless execution of secure and customized LoRa-based projects.

## 5. Conclusions

LoRa technology stands out as a leader and vanguard in its field, supported by an open-source community that provides access to information and developments relevant to this project. In turn, Blockly simplifies the task of creating specialized visual environments for programming. In addition, the ESP32 STEAMakers board streamlines system deployment and RFM9x module connection. The libraries for developing LoRa and LoRaWAN firmware and the Blockly environment are used, providing the necessary tools to generate Arduino-compatible code and the selected hardware through Arduinoblocks.

The main objective of bringing LoRa and LoRaWAN technology closer to the classroom, along with the development of low-cost nodes based on ESP32 and a visual tool for their programming, was achieved through prototyping the LoRa module for the ESP32 STEAMakers board. Connectivity and firmware development issues have been resolved through the visual tool based on Blockly and its integration with Arduinoblocks. Contributions include a working and reliable LoRa connectivity prototype, a visual programming environment for ESP32 devices, and practical examples in various application areas. These achievements allow educators and students to understand and experiment with the capabilities of ESP32 devices with LoRa connectivity.

As a limitation of this research and future work, the implementation of encryption and decryption functions is being developed to strengthen data security in LoRa communications. This ongoing effort seeks to ensure secure data exchange, making transmitted data incomprehensible without the decryption key, and will integrate with advanced LoRa communication settings on Arduinoblocks, providing users with a complete tool for secure and custom projects.

## Data Availability

All the materials developed in this work are freely available in the repository: https://github.com/arduinoblocks/lora/ (accessed on 25 August 2023).
